# The pathogenic and clinical characteristics of severe fever with thrombocytopenia syndrome patients with co-infections

**DOI:** 10.3389/fcimb.2023.1298050

**Published:** 2023-12-01

**Authors:** Huijuan Song, Siyu Zou, Yi Huang, Yun Wang, Ting Wang, Wei Wei, Ziyong Sun, Hongyan Hou

**Affiliations:** Department of Laboratory Medicine, Tongji Hospital, Tongji Medical College, Huazhong University of Science and Technology, Wuhan, China

**Keywords:** SFTS, co-infection, mortality, *Aspergillus fumigatus*, bacterial infection

## Abstract

**Objective:**

The study aimed to comprehensively describe and evaluate the pathogenic and clinical characteristics of severe fever with thrombocytopenia syndrome (SFTS) patients with co-infections.

**Methods:**

We retrospectively collected clinical data and laboratory indicators of the SFTS patients at Tongji Hospital from October 2021 to July 2023.

**Results:**

A total of 157 patients with SFTS virus (SFTSV) infection were involved in the analysis, including 43 co-infection and 114 non-co-infection patients. The pathogens responsible for co-infection were primarily isolated from respiratory specimens. Fungal infections, primarily *Aspergillus fumigatus*, were observed in 22 cases. Bacterial infections, with *Klebsiella pneumoniae* and carbapenem-resistant *Acinetobacter baumannii* as the main pathogens, were identified in 20 cases. SFTS patients with co-infection exhibited higher mortality (*P*=0.011) compared to non-co-infection patients. Among SFTS patients co-infected with both bacteria and fungi (8 cases) or specific drug-resistant strains (11 cases), the mortality rate was as high as 70% (14/19). In comparison with the non-co-infection group, SFTS patients with co-infection displayed significant alteration in inflammatory markers, coagulation function, and liver function indicators.

**Conclusion:**

The mortality rate of SFTS patients with co-infection is relatively high, underscoring the need for enhanced monitoring and timely, appropriate treatment to minimize the mortality rate.

## Introduction

Severe Fever with Thrombocytopenia Syndrome Virus (SFTSV) is an RNA virus that was first identified in China in 2010 and causes a clinical illness known as severe fever with thrombocytopenia syndrome (SFTS) (Yu et al., 2011; Zhang et al., 2012). Since its discovery, the incidence of SFTS has rapidly increased, posing a significant public health concern. The clinical manifestations of SFTS primarily include fever, diarrhea, and vomiting, and in severe cases, it can progress to multi-organ failure with a high mortality rate, ranging from 6% to 30% (Li et al., 2018; Casel et al., 2021; Li et al., 2021; Miao et al., 2021; Zhang X. et al., 2022).

Currently, the majority of studies have concentrated on predictive laboratory indicators for the occurrence and fatality of SFTS patients (Xu et al., 2018; Zhang et al., 2021; Liu et al., 2022; Zhang et al., 2023), with limited research integrating microbial infections into the assessment of risk factors for patient mortality. It has been reported that the mortality rate in SFTS patients was significantly increased when complicated with invasive pulmonary aspergillosis (IPA) (Chen et al., 2018; Bae et al., 2020; Hu et al., 2021; Xu et al., 2021). However, pathogens involved in co-infections, including *Aspergillus*, remain elusive (Ge et al., 2022; Zhang Y. et al., 2022), making the diagnosis of co-infections challenging and often leading to delayed treatment. Hence, it is crucial to describe the clinical characteristics of SFTS patients with concurrent infections and evaluate their risk factors, contributing to early diagnosis and timely administration of appropriate antibiotics, thereby reducing patient mortality. Some studies have evaluated laboratory parameters in SFTS patients to determine indicators that can be used for early prediction of IPA occurrence (Cui et al., 2013; Hu et al., 2021; Xu et al., 2021). Nevertheless, there is limited data on SFTS patients concurrently afflicted by bacterial infections. This study aimed to delineate the prevalence of SFTS patients with both bacterial and fungal infections, describe the characteristics of isolated pathogens, and subsequently evaluate the changes in clinical manifestations and laboratory indicators. The goal is to identify the risk factors associated with SFTS patients who are complicated by infections.

## Methods

### Study design

This retrospective cohort study was conducted at Tongji Hospital, Tongji Medical College, Huazhong University of Science and Technology, Wuhan, China, from October 2021 and July 2023. Patients were eligible for inclusion if SFTSV infection was confirmed positive by polymerase chain reaction or metagenomic next-generation sequencing (mNGS). Patients with immunosuppression, such as those undergoing transplant procedures or receiving long-term steroid therapy, were specifically excluded. Microorganisms were confirmed positive through cultivation, mNGS, (1, 3)-β-D-glucan (G), and galactomannan (GM) tests. Positive microbiological results were considered co-infections. The strains were identified by matrix-assisted laser desorption/ionization time-of-flight mass spectrometry (MALDI-TOF MS) (Autof ms1000, China) and mNGS. Antimicrobial susceptibility tests and results analysis were performed according to the standard of the Clinical and Laboratory Standards Institute (CLSI) (https://clsi.org/) M100 Ed33 guideline and the European Committee on Antimicrobial Susceptibility Testing (EUCAST) (https://www.eucast.org/).

### Data collection

Data was collected for each SFTS patient, including the following variables: (1) demographic (age, sex); (2) underlying diseases (hypertension, coronary disease, diabetes); (3) clinical symptoms (fever, gastrointestinal symptoms (diarrhea, nausea, vomiting, poor appetite, bellyache), disorders of consciousness); (4) laboratory data (laboratory indicators, mNGS results, microbiological culture, and drug sensitivity results); (5) treatment regimens (continuous renal replacement therapy (CRRT), and mechanical ventilation); (6) outcome: The survival time of the patient was defined as the interval between the onset of clinical symptoms (self-reported) and death, with a cut-off point of 28 days. Follow-up by phone call was used to determine outcomes for patients who have not reached the observation time during hospitalization.

### Statistical analysis

Continuous variables were presented as means ± standard deviation (SD) or median (interquartile range, IQR). Categorical variables were presented as numbers (%). Continuous variables were subject to the Wilcoxon rank-sum test or independent sample *t* test. The categorical variables are compared by Pearson Chi-square and Fisher’s Exact test between two groups. A p-value less than 0.05 was considered statistically significant. All variables with statistical significance in univariate analysis were included in multivariable logistic regression analysis for the model. The performance of various indicators was evaluated by the receiver operating characteristic (ROC) curve. Survival time data were analyzed using the Kaplan-Meier method and the log-rank test. The correlation between laboratory indicators was analyzed and visualized using the R package “corrplot”. Data were analyzed using SPSS version 25.0 (SPSS, Inc., Chicago, IL, USA), and plotted by the R 4.3.0 program (R Core Team).

## Results

### Demographics and clinical characteristics of SFTS patients

A total of 157 SFTSV-positive patients including 43 co-infection and 114 non-co-infection patients were enrolled in the analysis. As shown in **Table 1**, the average age of SFTS patients with co-infection was 63.140 ± 8.587 years old. Among the SFTS patients with co-infection, 28 (65.1%) were female. It was observed that the proportion of SFTS patients with hypertension was higher in the co-infection group compared to the non-co-infection group, and this difference was statistically significant (30/114 (26.3%) vs 20/43 (46.5%), *P*=0.015). However, there were no significant differences in terms of age, sex, and other underlying diseases between the co-infection and non-co-infection groups.

**Table 1:** Demographic and clinical characteristics of SFTS patients with and without co-infection.

Characteristics	Total cohort (n=157)	Group 0: Non-co-infection (n=114)	Group 1: Co-infection (n=43)	*P* value (Group 0 vs. 1)
Demographic characteristics				
Age, years	63.522 ± 9.534	63.667 ± 9.900	63.140 ± 8.587	0.758
Sex, female, %	91 (58.0%)	63 (55.3%)	28 (65.1%)	0.265
Underlying condition or illness				
Hypertension	50 (31.8%)	30 (26.3%)	20 (46.5%)	0.015 *
Coronary disease	12 (7.6%)	10 (8.8%)	2 (4.7%)	0.596
Diabetes	12 (7.6%)	8 (7.0%)	4 (9.3%)	0.886
Clinical symptoms				
Fever	146 (93.0%)	105 (92.1%)	41 (95.3%)	0.719
Gastrointestinal symptoms	86 (54.8%)	65 (57.0%)	21 (48.8%)	0.358
Disorders of consciousness	56 (35.7%)	32 (28.1%)	24 (55.8%)	0.001 **
Treatment regimens				
CRRT	48 (30.6%)	25 (21.9%)	23 (53.5%)	0.000 **
Mechanical ventilation	25 (15.9%)	10 (8.8%)	15 (34.9%)	0.000 **
Outcome				0.011 *
Survival	98 (62.4%)	78 (68.4%)	20 (46.5%)	
Death	59 (37.6%)	36 (31.6%)	23 (53.5%)	

SFTS, severe fever with thrombocytopenia syndrome; CRRT, continuous renal replacement therapy; *p<0.05; **p<0.01.

Our data showed that fever (93.0%) was the most frequent symptom in SFTS patients. Approximately 54.8% of SFTS patients experienced gastrointestinal symptoms, while 35.7% of SFTS patients showed signs of disordered consciousness (**Table 1**). Statistical analysis results revealed that the probability of disorder of consciousness in the co-infection group was significantly higher than that in the non-co-infection group. A higher proportion of patients in the co-infection group received the CRRT and mechanical ventilation treatment than that of the non-co-infection group. In addition, we found that 23 (53.5%) of the 43 SFTS patients complicated with co-infections died, while 78 (68.4%) of the 114 SFTS patients without co-infections survived, indicating that SFTS patients with co-infections have a significantly higher risk of death (**Table 1**).

### Species distribution and sample source of pathogens for co-infections

**Figure 1:**
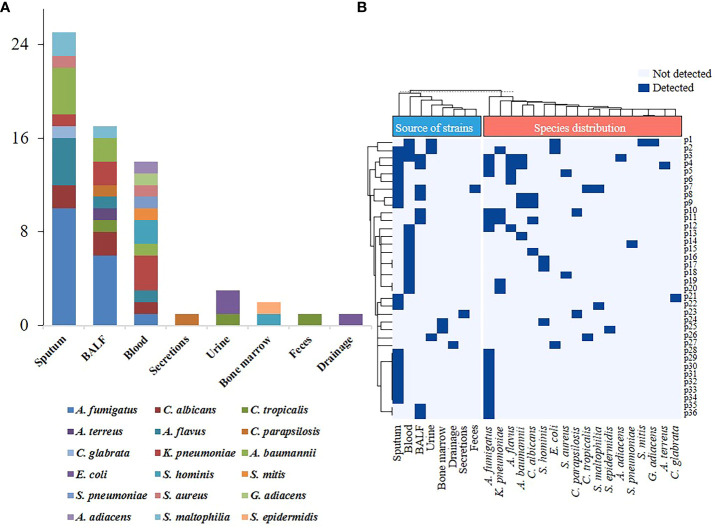
Species distribution and sample sources of pathogens isolated from severe fever with thrombocytopenia syndrome (SFTS) patients. **(A)** Sample sources of pathogens isolated from SFTS patients. **(B)** Sample types and pathogens distribution in SFTS patients with co-infection. BALF, bronchoalveolar lavage fluid; *A. fumigatus, Aspergillus fumigatus; C. albicans, Candida albicans; C. tropicalis, Candida tropicalis; A. terreus, Aspergillus terreus; A. flavus, Aspergillus flavus; C. parapsilosis; Candida parapsilosis; C. glabrata, Candida glabrata; K. pneumoniae, Klebsiella pneumoniae; A. baumannii, Acinetobacter baumannii; E. coli, Escherichia coli; S. hominis, Staphylococcus hominis; S. aureus, Staphylococcus aureus; S. epidermidis, Staphylococcus epidermidis; S. mitis, Streptococcus mitis; S. pneumoniae, streptococcus pneumoniae; G. adiacens, Granulicatella adiacens; A. adiacens, Abiotrophia adiacens; S. maltophilia, Stenotrophomonas maltophilia.*

Among 43 SFTS patients with co-infections, 7 patients were positive for GM test in serum, but negative in culture results (**Supplementary Table 1**). A total of 57 strains were isolated from other 36 SFTS patients with co-infections obtained from various sample sources. The distribution of pathogen types and sample sources of the isolates obtained from SFTS patients are depicted in **Figure 1**.

The pathogens were predominantly isolated from sputum samples (25/57, 43.9%) and bronchoalveolar lavage fluid (BALF) (17/57, 29.8%), followed by blood samples (14/57, 24.6%). Fungi accounted for the majority of isolated pathogens, with the highest isolation rate, observed for *Aspergillus fumigatus* (*A. fumigatus*) (15/57, 26.3%), followed by *Aspergillus flavus* (*A. flavus*) (5/57, 8.8%), and *Candida albicans* (*C. albicans*) (4/57, 7%). Among the cases of bacterial infection, *Klebsiella pneumoniae* (*K. pneumoniae*) and *Acinetobacter baumannii* (*A. baumannii*) were the most frequently identified pathogens, accounting for 6 out of 57 cases (10.5%) (**Supplementary Table 1**; **Figure 1A**).

Our results showed that 11 patients were simultaneously infected with multiple pathogens. Out of these 11 cases, 8 patients had co-infection of bacteria and fungi, with *A. fumigatus* being the most prevalent co-infecting pathogen. Specifically, one patient (Patient ID: p12) was infected with both *A. fumigatus* and *A. flavus*. The remaining two patients exhibited mixed bacterial infections. Patient p1 had *Granulicatella adiacens* and *Streptococcus mitis* isolated from blood samples, along with a urine sample that showed an *Escherichia coli* (*E. coli*) strain resistant to multiple drugs (**Figure 1B**; **Supplementary Table 1**). Patient p2 had simultaneous isolation of *K. pneumoniae* from both blood and sputum samples. Notably, *K. pneumoniae* isolated from the blood was susceptible to multiple antibiotics, while the sputum isolate exhibited multidrug resistance (MDR) (**Supplementary Table 1**). Additionally, this patient also had a multidrug-resistant *E. coli* isolated from the urine sample. According to the results of the antibiotic susceptibility tests, a total of three strains of the multidrug-resistant organism (MDRO), six strains of carbapenem-resistant *A. baumannii* (CRAB), two strains of carbapenem-resistant *K. pneumoniae* (CRKPN) and a strain of methicillin-resistant *Staphylococcus hominis* (MRSCN) were isolated (**Supplementary Table 1**). Moreover, two strains of CRAB were isolated from both sputum and BALF samples of the same patient (Patient ID: p4).

### Survival analysis of SFTS patients with co-infections

**Figure 2:**
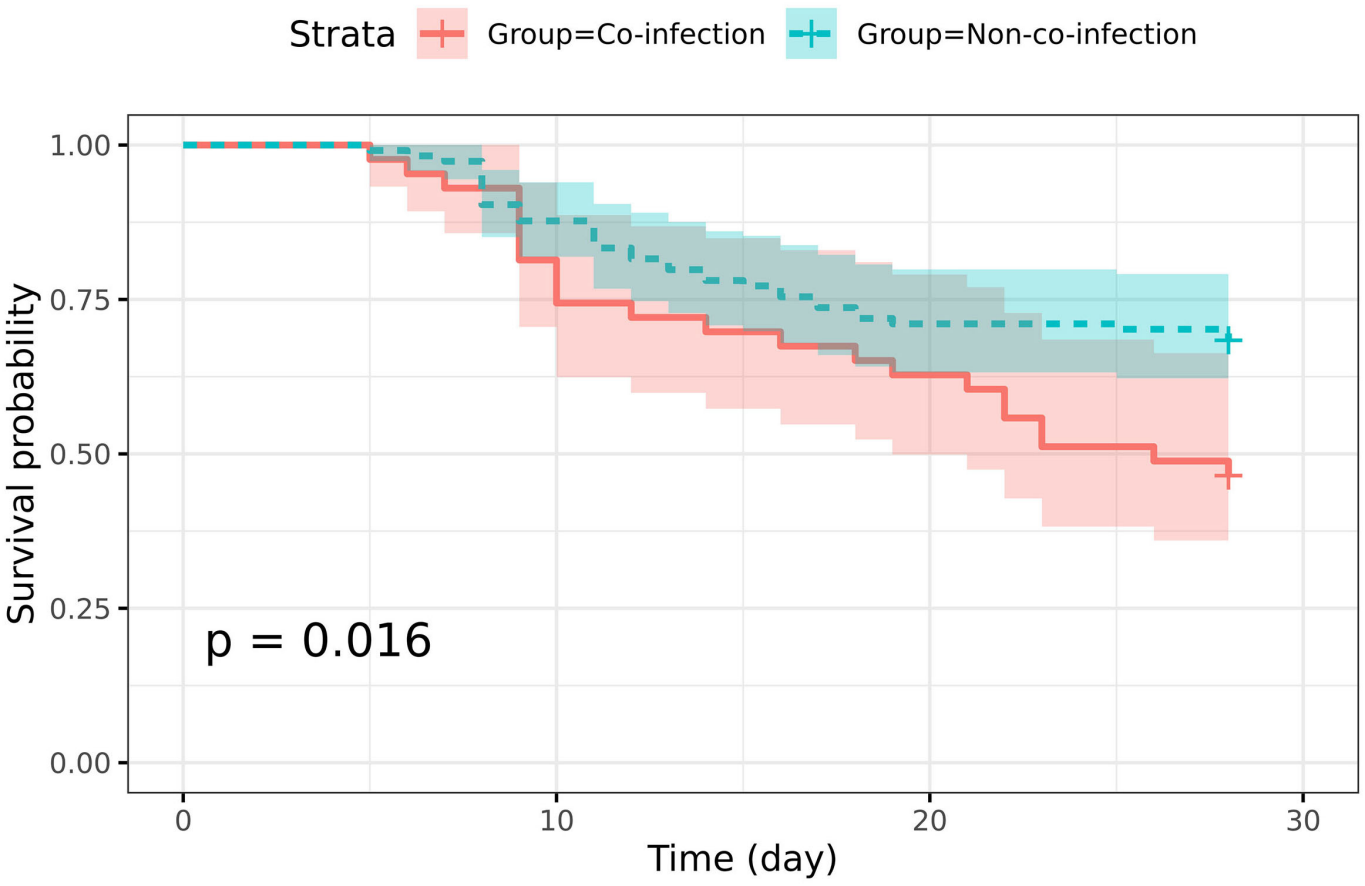
Survival analysis of severe fever with thrombocytopenia syndrome (SFTS) patients with and without co-infection. The Kaplan-Meier survival curve shows the 28-day survival rate of SFTS patients. The 95% confidence interval (CI) areas were displayed in different colors.

The Kaplan-Meier curve indicated a significant difference (*P*=0.016) in survival rates between the co-infection and non-co-infection groups of SFTS patients (**Figure 2**). In terms of pathogen type and resistance phenotype, there were no statistically significant differences observed between the two groups of SFTS patients with co-infections who survived and those who did not (**Supplementary Table 2**). However, the mortality rate of SFTS patients solely infected with bacteria (6/13, 46.2%) or fungi (7/15, 46.7%) was comparable, while SFTS patients infected with both bacteria and fungi experienced a higher mortality rate (6/8, 75.0%) (**Supplementary Table 2**). Furthermore, the mortality rate among patients infected with specific drug-resistant strains (MDRO, CRAB, CRKPN, MRSCN) reached as high as 72.7% (8/11), which was greater than the mortality rate for SFTS patients infected with drug-sensitive strains (11/25, 44.0%).

### Laboratory indicators for distinguishing between SFTS patients with and without co-infections

**Figure 3:**
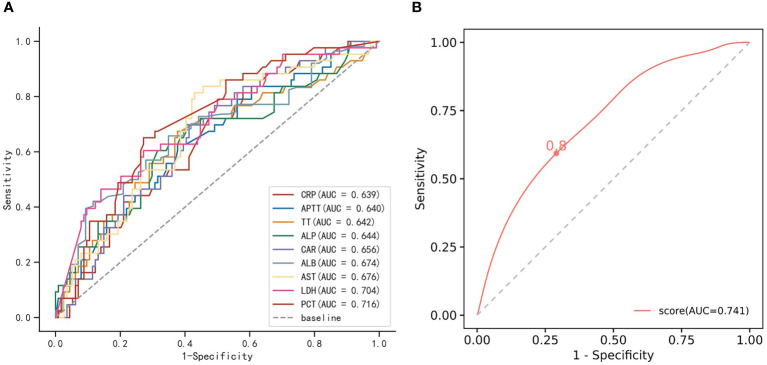
The receiver operating characteristic (ROC) curves. **(A)** The ROC curves of significant difference indicators in SFTS patients with co-infection and non-co-infection. **(B)** The ROC curve of the multivariable logistic regression model. The cutoff value is displayed on the graph. SFTS, severe fever with thrombocytopenia syndrome; AUC, area under the curve.

It was found that the level of albumin (ALB) was significantly lower in patients with co-infection than in those without co-infection (*P*=0.001) (**Table 2**). Additionally, there were statistically significant differences in several laboratory indicators, including aspartate aminotransferase (AST), alkaline phosphatase (ALP), lactate dehydrogenase (LDH), C-reactive protein (CRP), CRP-to-albumin ratio (CAR), procalcitonin (PCT), activated partial thromboplastin time (APTT) and thrombin time (TT), with higher levels in the co-infection group (**Table 2**). Furthermore, ROC curve analysis of these laboratory indicators showed that the AUC for most indicators ranged from 0.6 to 0.68. Notably, PCT and LDH exhibited higher AUC values, with PCT at 0.716 (95% CI, 0.629-0.802) and LDH at 0.704 (95% CI, 0.611-0.797) (**Supplementary Table 3**; **Figure 3A**). Specifically, when a threshold of 0.475 ng/mL was used, PCT showed a sensitivity of 65.1% and a specificity of 72.8% in distinguishing co-infection from non-co-infection patients. Similarly, LDH demonstrated a sensitivity of 60.5% and a specificity of 72.8% with a threshold of 1019.5 U/L (**Supplementary Table 3**). When the optimal threshold was selected, AST and CRP exhibited higher sensitivity (>70%) but lower specificity (<60%).

**Table 2:** Comparison of early laboratory indicators of severe fever with thrombocytopenia syndrome (SFTS) patients with and without co-infection.

Indicators	Group 1: Co-infection (n=43)	Group 0: Non-co-infection (n=114)	*P* value (Group 0 vs 1)
Routine indicators			
WBC (×10^9^/L)	3.640(2.405-7.290)	3.250(1.835-5.540)	0.287
NEU (×10^9^/L)	1.920(1.320-5.295)	2.035(1.055-4.107)	0.335
NEU (%)	70.700(56.900-80.850)	68.300(50.350-84.400)	0.883
LYM (%)	22.500(14.850-33.000)	22.600(11.375-36.650)	0.947
LYM (×10^9^/L)	1.039 ± 0.707	0.849 ± 0.666	0.118
MON (%)	8.274 ± 8.134	7.879 ± 6.741	0.758
MON (×10^9^/L)	0.240(0.100-0.390)	0.170(0.083-0.390)	0.371
RBC (×10^12^/L)	4.360(3.770-4.790)	4.070(3.812-4.380)	0.235
Hb (g/L)	124.000(112.500-144.500)	123.500(113.000-135.000)	0.594
PLT (×10^9^/L)	33.000(27.000-55.500)	43.500(31.000-58.750)	0.142
Liver function indicators			
ALT (U/L)	94.000(64.000-181.000)	74.500(48.000-133.000)	0.049 *
AST (U/L)	342.000(238.000-627.500)	189.000(100.250-347.750)	0.001 **
ALP (U/L)	90.000(64.500-166.500)	70.000(59.000-99.000)	0.006 **
GGT (U/L)	124.233 ± 145.836	87.140 ± 132.783	0.131
LDH (U/L)	1303.000(711.500-1867.000)	689.500(444.000-1244.750)	0.000 **
TP (g/L)	57.772 ± 6.458	60.213 ± 6.514	0.037 *
ALB (g/L)	29.135 ± 4.440	32.004 ± 4.820	0.001 **
GLB (g/L)	28.591 ± 4.871	28.210 ± 4.813	0.66
A/G	1.052 ± 0.262	1.167 ± 0.264	0.015 *
TBil (μmol/L)	7.600(5.950-13.400)	8.250(5.800-10.375)	0.537
DBil (μmol/L)	5.400(3.400-8.500)	4.300(2.600-6.300)	0.032 *
IBil (μmol/L)	2.800(1.800-4.300)	3.200(2.325-4.700)	0.116
TC (mmol/L)	2.880 ± 0.784	3.063 ± 0.791	0.197
Kidney function indicators			
Creatinine (μmol/L)	85.000(71.000-132.000)	80.500(66.000-106.000)	0.345
Uric acid (μmol/L)	254.000(184.400-392.250)	237.150(177.250-305.750)	0.17
Urea (mmol/L)	6.100(4.100-12.790)	6.155(4.310-8.355)	0.71
Inflammatory markers			
CRP (mg/L)	8.600(5.000-20.750)	5.750(1.675-10.650)	0.007 **
PCT (ng/mL)	0.620(0.350-1.800)	0.350(0.113-0.590)	0.000 **
CAR	0.292(0.175-0.794)	0.173(0.054-0.352)	0.003 **
NLR	2.949(1.520-5.491)	2.958(1.333-7.426)	0.901
NPR	0.045(0.033-0.126)	0.048(0.023-0.093)	0.101
SII	126.774(57.634-268.421)	128.213(46.703-369.353)	0.49
Coagulation function-related indicators			
PT (s)	13.000(12.550-14.100)	13.000(12.400-13.675)	0.245
PA (%)	102.173 ± 21.911	105.669 ± 23.482	0.398
APTT (s)	63.000(55.000-82.000)	56.500(46.050-65.500)	0.007 **
FIB (g/L)	2.818 ± 0.925	2.712 ± 0.850	0.497
D-dimer (μg/ml)	5.878 ± 5.557	5.256 ± 5.744	0.542
TT (s)	31.400(23.400-53.600)	24.050(21.000-32.925)	0.006 **
INR	0.970(0.925-1.050)	0.960(0.910-1.040)	0.365

*p<0.05; **p<0.01; WBC, white blood cell; NEU, neutrophil; LYM, lymphocyte; MON, monocyte; RBC, red blood cell; Hb, hemoglobin; PLT, platelet; ALT, alanine aminotransferase; AST, aspartate aminotransferase; ALP, alkaline phosphatase; GGT, γ-glutamyl transferase; LDH, lactate dehydrogenase; TP, total protein; ALB, albumin; GLB, globulin; A/G, albumin/globulin; TBil, total bilirubin; DBil, Direct bilirubin; IBil, Indirect bilirubin; TC, total cholesterol; CRP, C-reactive protein; PCT, procalcitonin; CAR, C-reactive protein-to-albumin ratio; NLR, neutrophil-to-lymphocyte ratio; NPR, neutrophil-to-platelet ratio; SII, systemic immune-inflammation index; PT, prothrombin time; PA, prothrombin activity; APTT, activated partial thromboplastin time; FIB, fibrinogen; TT, thrombin time; INR, international normalized ratio.

### The predictive ability of the combination of laboratory indicators for identifying SFTS patients with co-infection

A collinearity analysis of laboratory parameters is presented in **Supplementary Figure 1**. Considering the correlation between laboratory parameters and the integrity of laboratory data, ALP, ALB, and LDH were ultimately selected for multivariate regression analysis based on both clinical experience and professional knowledge. The results of the multivariate regression analysis indicated LDH as a significant risk factor for co-infection in SFTS patients (*P*<0.05). Notably, the diagnostic model developed exhibited moderate performance, with an AUC of 0.741. This performance was slightly better than using indicator LDH or PCT as single indicators (**Supplementary Table 4**; **Figure 3B**).

## Discussion

SFTS is a life-threatening disease that has garnered global attention. Co-infections can exacerbate the condition, leading to multiple organ dysfunction and higher mortality rates. Therefore, it is crucial to diagnose co-infection in SFTS patients promptly and implement effective treatment strategies. The early identification of risk factors for SFTS co-infection is very important.

Currently, there is limited research that describes the results of microbiological culture in SFTS patients with co-infection. A study by Yin Zhang and colleagues evaluated the incidence and treatment regimes of SFTS patients with microbial infections but did not provide detailed information about the strains or compare laboratory indicators between co-infection and non-co-infection groups (Zhang Y. et al., 2022). In this study, we offered a comprehensive description of the pathogens isolated from SFTS patients with microbial infections, including the types of pathogens, sample sources, and antimicrobial susceptibility results. We found a high incidence of microbial infections in SFTS patients, with respiratory tract fungal infections being more common than bacterial infections. *A. fumigatus* and *A. flavus* were the most frequently isolated fungi in SFTS patients, consistent with previous reports (Zhang Y. et al., 2022). Additionally, we observed that the most frequently isolated pathogens in SFTS patients with bacterial infections were CRAB and *K. pneumoniae*. Notably, CRAB, *Aspergillus terreus*, and *Abiotrophia adiacens* were previously unreported in SFTS patients with co-infections.

Previous research indicated that SFTS patients with fungus infections have a higher mortality rate (Li et al., 2018; Xu et al., 2021; Zhang et al., 2021). Similarly, we found that the mortality rate of SFTS patients with microbial infections was significantly higher than that of the non-co-infection group. Our findings revealed an increasing trend in the mortality rate among SFTS patients who were concurrently infected with both bacteria and fungi or infected with specific drug-resistant strains. However, this trend did not reach statistical significance, which may be attributed to the relatively small number of cases available for analysis. Our results suggested that the type of pathogen and the antibiotic susceptibility phenotype of the strain had a certain impact on the prognosis of SFTS patients, emphasizing the importance of microbiological monitoring and identification of drug sensitivity phenotypes.

Neurological symptoms in SFTS patients have been identified as an important predictor of fatal outcomes (Guo et al., 2016). In our study, the SFTS patients with co-infections were more likely to have neurological symptoms and have a higher mortality rate. To determine the predictive indicators of co-infection in SFTS patients, we further compared the differences in laboratory indicators between co-infection and non-co-infection groups. The results showed that the indicators with differences between the two groups were mainly inflammatory markers (CRP, CAR, PCT, IL-6), coagulation function-related indicators (TT, APTT), and liver function-related indicators (ALB, AST, LDH, ALP, etc.). Liver injury is common in SFTS patients and is associated with patient mortality (Lu et al., 2022; Zhao et al., 2022). Previous studies found that ALT and ALP are independent risk factors for mortality in SFTS patients (Li et al., 2018; Xu et al., 2021; Zhang et al., 2021). Based on this, we believe that microbial infections in SFTS patients mainly exacerbate liver damage, thereby increasing mortality. Thus, it is necessary to closely monitor SFTS patient symptoms and evaluate laboratory indicators, especially liver function indices.

This study offered a comprehensive description of the epidemiological and clinical features of microbial infections in SFTS patients, highlighting the complexities of co-infections in SFTS patients. Additionally, potential predictive factors for co-infections in SFTS patients were identified through laboratory indicators analysis, emphasizing the importance of considering these factors in clinical management. However, our study has the following limitations. Firstly, the microbiological testing results were not validated using multiple methods, which could have potentially led to inaccurate infection rates. Secondly, due to the limited number of cases and incomplete laboratory data, certain important clinical indicators were excluded from the prediction model, potentially impacting its accuracy and predictive power.

## Conclusion

In conclusion, this study provided valuable Supplementary Data on SFTS patients with co-infections, contributing to the epidemiological investigation of SFTSV infection and offering insights for improved disease management and reduced incidence rate and mortality. Future research should focus on expanding the sample size and incorporating additional laboratory indicators to further investigate the risk factors for co-infection in SFTS patients and optimize diagnostic models.

## Data availability statement

The original contributions presented in the study are included in the article/**Supplementary Material**. Further inquiries can be directed to the corresponding author.

## Ethics statement

The studies involving humans were approved by the Medical Ethics Committee of Tongji Hospital. The studies were conducted in accordance with the local legislation and institutional requirements. The ethics committee/institutional review board waived the requirement of written informed consent for participation from the participants or the participants’ legal guardians/next of kin because the research has been approved by the Medical Ethics Committee of Tongji Hospital. The committee also approved the abandonment of the informed consent form for participation in this study due to its retrospective design.

## Author contributions

HS: Data curation, Formal Analysis, Investigation, Methodology, Software, Validation, Visualization, Writing – original draft, Writing – review & editing. SZ: Methodology, Writing – review & editing. YH: Methodology, Writing – review & editing. YW: Investigation, Writing – review & editing. TW: Investigation, Writing – review & editing. WW: Investigation, Methodology, Writing – review & editing. ZS: Funding acquisition, Investigation, Writing – review & editing. HH: Formal Analysis, Investigation, Methodology, Resources, Writing – review & editing.

## Supplementary material

Supplementary Figure 1 The collinearity analysis of significant difference indicators in severe fever with thrombocytopenia syndrome (SFTS) patients. PCT, procalcitonin; LDH, lactate dehydrogenase; AST, aspartate aminotransferase; ALB, albumin; ALP, alkaline phosphatase; TT, thrombin time; APTT, activated partial thromboplastin time; CRP, C-reactive protein; CAR, C-reactive protein-to-albumin ratio.

## References

[B1] Bae S. Hwang H. J. Kim M. Y. Kim M. J. Chong Y. P. Lee S.. (2020). Invasive pulmonary aspergillosis in patients with severe fever with thrombocytopenia syndrome. Clin. Infect. Dis. 70 (7), 1491–1494. doi: 10.1093/cid/ciz673 31342053

[B2] Casel M. A. Park S. J. Choi Y. K. (2021). Severe fever with thrombocytopenia syndrome virus: Emerging novel phlebovirus and their control strategy. Exp. Mol. Med. 53 (5), 713–722. doi: 10.1038/s12276-021-00610-1 33953322 PMC8178303

[B3] Chen X. Yu Z. Qian Y. Dong D. Hao Y. Liu N.. (2018). Clinical features of fatal severe fever with thrombocytopenia syndrome that is complicated by invasive pulmonary aspergillosis. J. Infect. Chemother. 24, 422–427. doi: 10.1016/j.jiac.2018.01.005 29428567

[B4] Cui N. Wang H. Long Y. Liu D. (2013). CD8^+^ T-cell counts: An early predictor of risk and mortality in critically ill immunocompromised patients with invasive pulmonary aspergillosis. Crit. Care (London England). 17 (4), R157. doi: 10.1186/cc12836 PMC4057447 23883548

[B5] Ge H. Wang G. Guo P. Zhao J. Zhang S. Xu Y.. (2022). Coinfections in hospitalized patients with severe fever with thrombocytopenia syndrome: A retrospective study. J. Med. Virol. 94 (12), 5933–5942. doi: 10.1002/jmv.28093 36030552

[B6] Guo C. Lu Q. Ding S. Hu C. Hu J. Wo Y.. (2016). Epidemiological and clinical characteristics of severe fever with thrombocytopenia syndrome (SFTS) in China: An integrated data analysis. Epidemiol. Infect. 144 (6), 1345–1354. doi: 10.1017/S0950268815002678 26542444

[B7] Hu L. Kong Q. Yue C. Xu X. Xia L. Bian T.. (2021). Early-Warning immune predictors for invasive pulmonary aspergillosis in severe patients with severe fever with thrombocytopenia syndrome. Front. Immunol. 12. doi: 10.3389/fimmu.2021.576640 PMC8138034 34025635

[B8] Li J. Li S. Yang L. Cao P. Lu J. (2021). Severe fever with thrombocytopenia syndrome virus: A highly lethal bunyavirus. Crit. Rev. Microbiol. 47 (1), 112–125. doi: 10.1080/1040841X.2020.1847037 33245676

[B9] Li H. Lu Q. Xing B. Zhang S. Liu K. Du J.. (2018). Epidemiological and clinical features of laboratory-diagnosed severe fever with thrombocytopenia syndrome in China 2011-17: a prospective observational study. Lancet Infect. diseases. 18 (10), 1127–1137. doi: 10.1016/S1473-3099(18)30293-7 30054190

[B10] Liu Z. Zhang R. Zhou W. Ma R. Han L. Zhao Z.. (2022). High levels of C-reactive protein-to-albumin ratio (CAR) are associated with a poor prognosis in patients with severe fever with thrombocytopenia syndrome in early stage. J. Med. Virol. 94 (11), 5375–5384. doi: 10.1002/jmv.27972 35790466 PMC9540880

[B11] Lu S. Xu L. Liang B. Wang H. Wang T. Xiang T.. (2022). Liver function derangement in patients with severe fever and thrombocytopenia syndrome. J. Clin. Trans. hepatology. 10 (5), 825–834. doi: 10.14218/JCTH.2021.00345 PMC9547257 36304508

[B12] Miao D. Liu M. Wang Y. Ren X. Lu Q. Zhao G.. (2021). Epidemiology and ecology of severe fever with thrombocytopenia syndrome in China 2010-2018. Clin. Infect. Dis. 73 (11), e3851–e3858. doi: 10.1093/cid/ciaa1561 33068430 PMC8664468

[B13] Xu Y. Shao M. Liu N. Tang J. Gu Q. Dong D. (2021). Invasive pulmonary aspergillosis is a frequent complication in patients with severe fever with thrombocytopenia syndrome: A retrospective study. Int. J. Infect. Dis. 105, 646–652. doi: 10.1016/j.ijid.2021.02.088 33640568

[B14] Xu X. Sun Z. Liu J. Zhang J. Liu T. Mu X.. (2018). Analysis of clinical features and early warning indicators of death from severe fever with thrombocytopenia syndrome. Int. J. Infect. Dis. 73, 43–48. doi: 10.1016/j.ijid.2018.05.013 29859247

[B15] Yu X. Liang M. Zhang S. Liu Y. Li J. Sun Y.. (2011). Fever with thrombocytopenia associated with a novel bunyavirus in China. New Engl. J. Med. 364 (16), 1523–1532. doi: 10.1056/NEJMoa1010095 21410387 PMC3113718

[B16] Zhang Y. He Y. Dai Y. Xiong Y. Zheng H. Zhou D.. (2012). Hemorrhagic fever caused by a novel Bunyavirus in China: Pathogenesis and correlates of fatal outcome. Clin. Infect. Dis. 54 (4), 527–533. doi: 10.1093/cid/cir804 22144540

[B17] Zhang Y. Huang Y. Xu Y. (2022). Associated microbiota and treatment of severe fever with thrombocytopenia syndrome complicated with infections. J. Med. Virol. 94 (12), 5916–5921. doi: 10.1002/jmv.28059 35945160

[B18] Zhang Y. Miao W. Xu Y. Huang Y. (2021). Severe fever with thrombocytopenia syndrome in Hefei: Clinical features, risk factors, and ribavirin therapeutic efficacy. J. Med. Virol. 93 (6), 3516–3523. doi: 10.1002/jmv.26544 32965706

[B19] Zhang X. Zhao C. Cheng C. Zhang G. Yu T. Lawrence K.. (2022). Rapid spread of severe fever with thrombocytopenia syndrome virus by parthenogenetic asian longhorned ticks. Emerg. Infect. Dis. 28 (2), 363–372. doi: 10.3201/eid2802.211532 35075994 PMC8798674

[B20] Zhang Y. Zhong P. Wang L. Zhang Y. Li N. Li Y.. (2023). Development and validation of a clinical risk score to predict the occurrence of critical illness in hospitalized patients with SFTS. J. infection Public Health 16 (3), 393–398. doi: 10.1016/j.jiph.2023.01.007 36706468

[B21] Zhao J. Ge H. Wang G. Lin L. Yuan Y. Xu Y.. (2022). Fatal patients with severe fever with thrombocytopenia syndrome in China. Int. J. Infect. Dis. 125, 10–16. doi: 10.1016/j.ijid.2022.10.008 36241165

