# Supplementary material for: The pathogenic and clinical characteristics of severe fever with thrombocytopenia syndrome patients with co-infections
Source: Front Cell Infect Microbiol. 2023 Dec 1;13:1298050. doi: 10.3389/fcimb.2023.1298050 (PMC10722497; doi:10.3389/fcimb.2023.1298050)
Supplement: Supplementary file 2 [file Table_2.docx]

| **Supplementary Table 2**. The types of infection and drug sensitivity phenotypes between the death and survival groups of SFTS patients with co-infection. | | | | |
| --- | --- | --- | --- | --- |
| **Characteristics** | **Total cohort (n=36)** | **Survival (n=17)** | **Death (n=19)** | ***P* value (Survival vs. Death)** |
| **Types of infection** |  |  |  | 0.435 |
| Bacterial infection | 13 (36.1%) | 7 (41.2%) | 6 (31.6%) |  |
| Fungal infection | 15 (41.7%) | 8 (47.1%) | 7 (36.8%) |  |
| Bacterial and fungal infection | 8 (22.2%) | 2 (11.8%) | 6 (31.6%) |  |
| **Drug resistance type** |  |  |  | 0.156 |
| Sensitive strains | 25 (69.4%) | 14 (82.4%) | 11 (57.9%) |  |
| Resistant strains | 11 (30.6%) | 3 (17.6%) | 8 (42.1%) |  |
| CRAB | 5 (13.9%) | 1 (5.9%) | 4 (21.1%) |  |
| MDRO | 3 (8.3%) | 1 (5.9%) | 2 (10.5%) |  |
| CRKPN | 2 (5.6%) | 0 (0%) | 2 (10.5%) |  |
| MRSCN | 1 (2.8%) | 1 (5.9%) | 0 (0%) |  |
| SFTS, severe fever with thrombocytopenia syndrome; MDRO, multidrug-resistant organism; CRAB, carbapenem-resistant *Acinetobacter baumannii*; CRKPN, carbapenem-resistant *Klebsiella pneumoniae*; MRSCN, methicillin-resistant *Staphylococcus hominis.* | | | | |
