# Supplementary material for: The pathogenic and clinical characteristics of severe fever with thrombocytopenia syndrome patients with co-infections
Source: Front Cell Infect Microbiol. 2023 Dec 1;13:1298050. doi: 10.3389/fcimb.2023.1298050 (PMC10722497; doi:10.3389/fcimb.2023.1298050)
Supplement: Supplementary file 3 [file Table_3.docx]

| **Supplementary Table 3.** The performance of Laboratory indexes for distinguishing between co-infection and non-co-infection SFTS patients. | | | | |
| --- | --- | --- | --- | --- |
| **Variable** | **AUC (95% CI)** | **Cutoff value** | **Sensitivity** | **Specificity** |
| PCT | 0.716 (0.629-0.802) | 0.475 | 65.1% | 72.8% |
| LDH | 0.704 (0.611-0.797) | 1019.5 | 60.5% | 72.8% |
| AST | 0.676 (0.584-0.769) | 210.5 | 81.4% | 57.0% |
| ALB | 0.674 (0.583-0.764) | 30.05 | 65.1% | 65.8% |
| CAR | 0.656 (0.565-0.746) | 0.216 | 69.8% | 58.8% |
| ALP | 0.644 (0.542-0.745) | 75.5 | 69.8% | 59.6% |
| TT | 0.642 (0.541-0.744) | 25.95 | 67.4% | 62.3% |
| APTT | 0.640 (0.543-0.738) | 59.15 | 62.8% | 62.3% |
| CRP | 0.639 (0.547-0.732) | 6.125 | 72.1% | 52.6% |
| SFTS, severe fever with thrombocytopenia syndrome; AUC, area under the curve; CI, confidence interval; PCT, procalcitonin; LDH, lactate dehydrogenase; AST, aspartate aminotransferase; ALB, albumin; CAR, C-reactive protein-to-albumin ratio; ALP, alkaline phosphatase; TT, thrombin time; APTT, activated partial thromboplastin time; CRP, C-reactive protein. | | | | |
