# Supplementary material for: The pathogenic and clinical characteristics of severe fever with thrombocytopenia syndrome patients with co-infections
Source: Front Cell Infect Microbiol. 2023 Dec 1;13:1298050. doi: 10.3389/fcimb.2023.1298050 (PMC10722497; doi:10.3389/fcimb.2023.1298050)
Supplement: Supplementary file 4 [file Table_4.docx]

| **Supplementary Table 4**. Multivariate logistic regression analysis for co-infection in SFTS patients. | | | |
| --- | --- | --- | --- |
| **Variable** | **β** | ***P* value** | **OR (95%CI)** |
| LDH | -0.001 | 0.027 * | 0.999 (0.998-1.000) |
| ALB | 0.066 | 0.172 | 1.068 (0.972-1.175) |
| ALP | -0.004 | 0.074 | 0.996 (0.991-1.000) |
| SFTS, severe fever with thrombocytopenia syndrome; LDH, lactate dehydrogenase; ALB, albumin; ALP, alkaline phosphatase; β, regression coefficient; OR, Odds ratio; CI, confidence interval; * *p*<0.05. | | | |
