# Supplementary figures and images for: The pathogenic and clinical characteristics of severe fever with thrombocytopenia syndrome patients with co-infections
Source: Front Cell Infect Microbiol. 2023 Dec 1;13:1298050. doi: 10.3389/fcimb.2023.1298050 (PMC10722497; doi:10.3389/fcimb.2023.1298050)

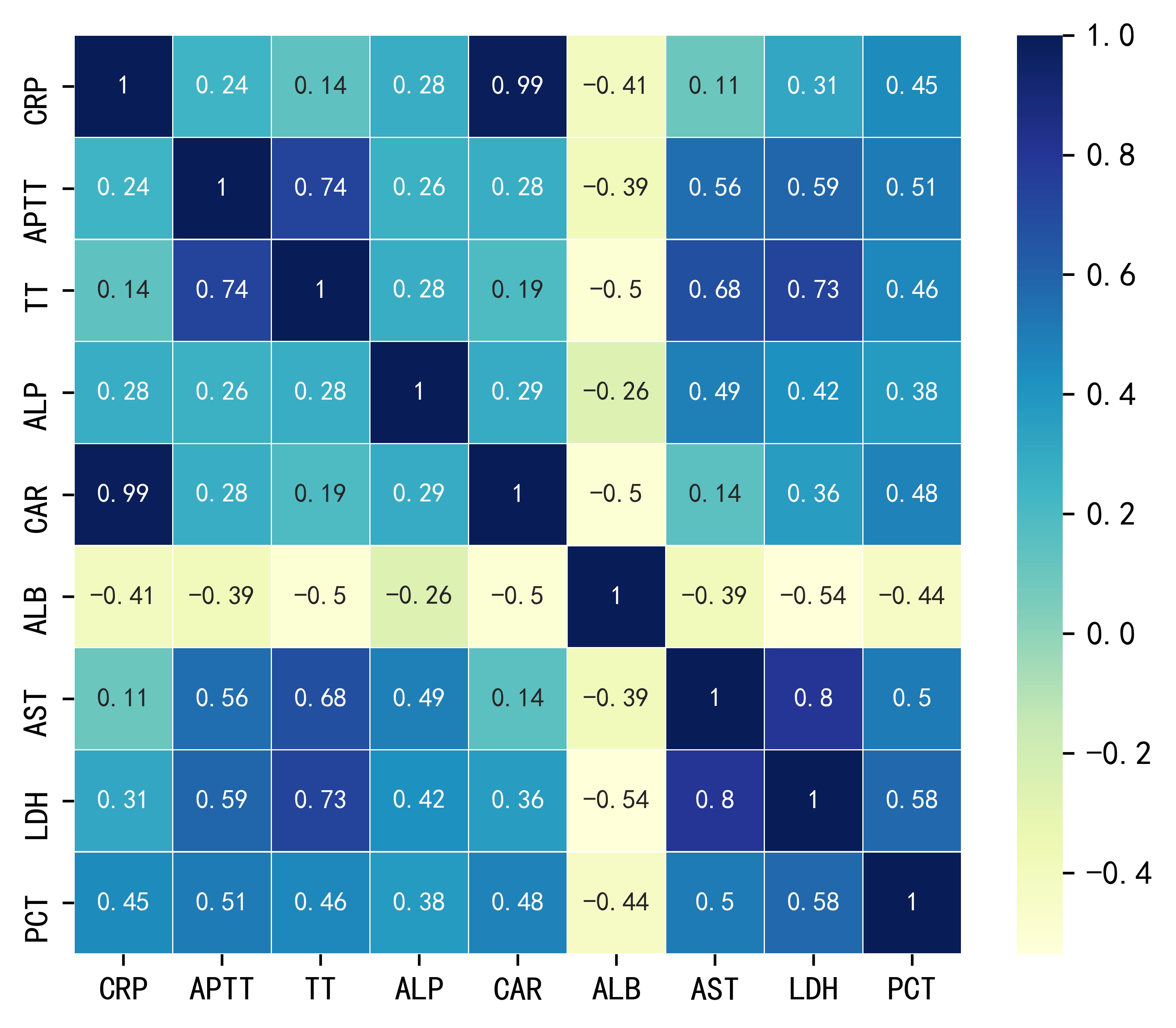

Supplement: Supplementary Figure 1 — The collinearity analysis of significant difference indicators in severe fever with thrombocytopenia syndrome (SFTS) patients. PCT, procalcitonin; LDH, lactate dehydrogenase; AST, aspartate aminotransferase; ALB, albumin; ALP, alkaline phosphatase; TT, thrombin time; APTT, activated partial thromboplastin time; CRP, C-reactive protein; CAR, C-reactive protein-to-albumin ratio. [file Image_1.jpeg]
